# Effects of Pyridoxine Deficiency on Hippocampal Function and Its Possible Association with V-Type Proton ATPase Subunit B2 and Heat Shock Cognate Protein 70

**DOI:** 10.3390/cells9051067

**Published:** 2020-04-25

**Authors:** Hyo Young Jung, Woosuk Kim, Kyu Ri Hahn, Hyun Jung Kwon, Sung Min Nam, Jin Young Chung, Yeo Sung Yoon, Dae Won Kim, Dae Young Yoo, In Koo Hwang

**Affiliations:** 1 Department of Anatomy and Cell Biology, College of Veterinary Medicine, and Research Institute for Veterinary Science, Seoul National University, Seoul 08826, Korea; hyoyoung@snu.ac.kr (H.Y.J.); hkinging@snu.ac.kr (K.R.H.); ysyoon@snu.ac.kr (Y.S.Y.); 2 Department of Biomedical Sciences, and Research Institute for Bioscience and Biotechnology, Hallym University, Chuncheon 24252, Korea; tank3430@hallym.ac.kr; 3 Department of Biochemistry and Molecular Biology, Research Institute of Oral Sciences, College of Dentistry, Gangneung-Wonju National University, Gangneung 25457, Korea; donuts25@gwnu.ac.kr (H.J.K.); kimdw@gwnu.ac.kr (D.W.K.); 4 Department of Anatomy, College of Veterinary Medicine, Konkuk University, Seoul 05030, Korea; lovingvet@gmail.com; 5 Department of Veterinary Internal Medicine and Geriatrics, College of Veterinary Medicine, Kangwon National University, Chuncheon 24341, Korea; jychung77@gmail.com; 6 Department of Anatomy, College of Medicine, Soonchunhyang University, Cheonan 31151, Korea

**Keywords:** pyridoxine deficiency, novel object recognition memory, neurogenesis, V-type proton ATPase subunit B2, heat shock cognate protein 70

## Abstract

Pyridoxine, one of the vitamin B_6_ vitamers, plays a crucial role in amino acid metabolism and synthesis of monoamines as a cofactor. In the present study, we observed the effects of pyridoxine deficiency on novel object recognition memory. In addition, we examined the levels of 5-hydroxytryptamine (5-HT), 5-hydroxyindoleacetic acid (5-HIAA), 3,4-dihydroxyphenethylamine (DA), 3,4-dihydroxyphenylacetic acid, and homovanillic acid and the number of proliferating cells and neuroblasts in the hippocampus. We also examined the effects of pyridoxine deficiency on protein profiles applying a proteomic study. Five-week-old mice fed pyridoxine-deficient diets for 8 weeks and showed a significant decrease in the serum and brain (cerebral cortex, hippocampus, and thalamus) levels of pyridoxal 5′-phosphate, a catalytically active form of vitamin-B_6_, and decline in 5-HT and DA levels in the hippocampus compared to controls fed a normal chow. In addition, pyridoxine deficiency significantly decreased Ki67-positive proliferating cells and differentiated neuroblasts in the dentate gyrus compared to controls. A proteomic study demonstrated that a total of 41 spots were increased or decreased more than two-fold. Among the detected proteins, V-type proton ATPase subunit B2 (ATP6V1B2) and heat shock cognate protein 70 (HSC70) showed coverage and matching peptide scores. Validation by Western blot analysis showed that ATP6V1B2 and HSC70 levels were significantly decreased and increased, respectively, in pyridoxine-deficient mice compared to controls. These results suggest that pyridoxine is an important element of novel object recognition memory, monoamine levels, and hippocampal neurogenesis. Pyridoxine deficiency causes cognitive impairments and reduction in 5-HT and DA levels, which may be associated with a reduction of ATP6V1B2 and elevation of HSC70 levels in the hippocampus.

## 1. Introduction

In the human brain, very few regions have the capacity to produce new neurons throughout life [1]. Among these regions, the subgranular zone of the hippocampal dentate gyrus retains the capacity; the newly generated cells move to the granule cell layer and become mature granule cells in the dentate gyrus [1,2]. In addition, the hippocampus forms part of the limbic system and plays an important role in memory acquisition/consolidation and spatial navigation [3]. GABAergic interneurons in the polymorphic layer of the dentate gyrus play important roles in regulating neurogenesis by innervating to granule cell lineage populations [1,4]. Newly generated neurons by adult hippocampal neurogenesis are closely related to various memory processes including novel object recognition memory [5].

Pyridoxine, one of the vitamin B_6_ vitamers, is an essential dietary nutrient for mammals because it is not synthesized in mammalian cells [6]. It is absorbed in the intestine, transported into the liver and transformed into pyridoxal 5′-phosphate (PLP), which is the active form of pyridoxine. PLP is utilized as a cofactor in more than 140 biochemical reactions by many enzymes related to amino acid metabolism [7,8]. Specifically, PLP is used as a cofactor during the synthesis of serotonin (5-hydroxytryptamine, 5-HT) and dopamine (3,4-dihydroxyphenethylamine, DA), which are related to depression, anxiety, and hippocampal neurogenesis [9,10,11,12,13]. PLP is an essential element during fetal and postnatal development, as it controls the regulation of GABA levels in the brain [14]. During myelination, PLP also serves as a cofactor and is also an important cofactor in sphingolipid synthesis and myelin formation [15,16].

Several lines of evidence demonstrate that pyridoxine has positive effects against neurological disorders including brain contusion, ischemia, and triton-induced neurotoxicity [17,18,19,20]. In addition, pyridoxine protects neurons from Parkinson’s disease by facilitating the dimerization of PKM2 to promote glutathione biosynthesis [21]. The knockout of PLP phosphatase, a degradation enzyme of PLP, improves the cognitive function of mice [22]. In addition, previous studies demonstrate that pyridoxine treatment significantly increases hippocampal neurogenesis in mice [10,12], while pyridoxine deficiency causes growth retardation [13,23] and abnormalities in structures and electrical activity in the hippocampus [24]. However, there are few reports about the effects of pyridoxine deficiency on hippocampal functions, especially the novel object recognition, serotonin, and hippocampal neurogenesis.

In the present study, we investigated the effects of pyridoxine-deficient diets on novel object memory, serotonin turnover, proliferating cells, and immature neuroblasts in the hippocampus. In addition, we tried to identify the significantly changed protein expression levels in the hippocampus under pyridoxine deficiency based on two dimensional gel electrophoresis (2DE) followed by matrix-assisted laser desorption/ionization time-of-flight mass spectrometry (MALDI-TOF MS) in the hippocampus.

## 2. Materials and Methods

### 2.1. Experimental Animals

Male C57BL/6J mice (4 weeks of age) were purchased from Jackson Laboratory Co. Ltd. (Bar Harbor, ME, USA). The handling and care of the animals conformed to the Guide for the Care and Use of Laboratory Animals (8th edition, 2011). Experimental protocols were approved by the Institutional Animal Care and Use Committee (IACUC) of Seoul National University (SNU-190408-2).

After 1 week of acclimation, mice were fed with AIN-76A diet (D15501R, control, Research Diets, NJ, USA) and pyridoxine-deficient (Pyr-def) diet (D10001, Research Diets) for 8 weeks. This schedule was chosen because the half-life elimination of pyridoxine exceeds 15 to 20 days [25].

### 2.2. Novel Object Recognition Test

The novel object recognition test was conducted to elucidate the effects of pyridoxine deficiency on recognition memory in mice as previously described [10]. To avoid experimental errors induced by odor in certain places, the floor was covered with woodchip bedding and the objects were cleaned with bleach to remove residual odors. Briefly, on day 55 (training day) of diet feeding, mice were trained in the open box (25 cm × 25 cm × 25 cm) made of black acryl and were allowed to explore the apparatus for 2 min. On day 56 (testing day), mice were placed to search two identical objects located in opposite corners of the apparatus for 2 min. After 1 h interval, mice were replaced in the box to explore one identical (familiar) and one new object. Exploration time was measured when mice directed the nose toward the object at a distance of no more than 2 cm and/or touching the object with the nose. The procedure was recorded with a digital camera system (Basler 106200, Ahrensburg, Germany) and the data was analyzed by Ethovision XT14 (Wageningen, The Netherlands).

The distinction between familiar and new in the testing day was determined by comparing the time spent exploring the familiar object with that spent exploring the new object. The discrimination index represents the difference in exploration time expressed as a proportion of the total time spent exploring the two objects in the testing trial.

### 2.3. High-Performance Liquid Chromatography Analysis

To examine the effects of pyridoxine deficiency on PLP levels in the serum and brain (cerebral cortex, hippocampus, and thalamus), as well as serotonin and DA metabolism in the hippocampus, high-performance liquid chromatography (HPLC) for PLP, 5-HT, 5-hydroxyindoleacetic acid (5-HIAA), DA, 3,4-dihydroxyphenylacetic acid (DOPAC), and homovanillic acid (HVA) was conducted, as described before [10,26]. Briefly, animals (*n* = 7 per group) were anesthetized with a mixture of alfaxalone (Alfaxan, 75 mg/kg; Careside, Seongnam, South Korea) and xylazine (10 mg/kg; Bayer Korea, Seoul, South Korea) and the blood and brain (cerebral cortex, hippocampus, and thalamus) were collected. Serum and an aliquot of processed homogenate were injected onto a C_18_ reverse-phase column (250 mm × 4.6 mm, 5 μm; Agilent Technologies, Santa Clara, CA, USA) in an HPLC system (Agilent 1100 series, Agilent Technologies) equipped with an electrochemical detector. The mobile phase (0.1 M acetate-citrate buffer with 17% methanol) allowed for the separation of 5-HT and its metabolite, 5-HIAA [27]. The turnover ratios of 5-HIAA to 5-HT and DA to DOPAC+HVA are considered an index of cell activity that results in the release, reuptake, and metabolism of 5-HT to 5-HIAA and DA to HVA.

### 2.4. Immunohistochemistry

To visualize the proliferating cells and differentiated neuroblasts in the hippocampus, immunohistochemical staining was conducted for Ki67 and doublecortin (DCX), respectively, as previously described [9]. Briefly, animals (*n* = 5 per group) were anesthetized with a mixture of 75 mg/kg alfaxalone and 10 mg/kg xylazine at 8 weeks after diet feeding and were perfused transcardially [10]. Frozen brain sections (30-μm thickness) were collected into six-well plates based on the mouse atlas by Franklin and Paxinos [28] between 1.46 mm and 2.46 mm posterior to the bregma.

Five sections located 90 μm apart were used for immunohistochemical staining with each antibody [10]; sections were incubated with rabbit anti-Ki67 antibody (1:1000, Abcam, Cambridge, UK), and a rabbit anti-DCX antibody (1:5000, Abcam) and the immunoreaction was visualized with nickel intensified 3,3′-diaminobenzidine tetrachloride (Sigma, St. Louis, MO, USA) in 0.1 M Tris-HCl buffer (pH 7.2). Sections were dehydrated and mounted on gelatin-coated slides in Canada balsam (Kanto Chemical, Tokyo, Japan).

### 2.5. Proteomic Analysis

#### 2.5.1. Protein Preparation for 2DE

Animals (*n* = 20 in each group) were anesthetized with a mixture of alfaxalone and xylazine at 8 weeks after diet feeding and the hippocampus was isolated from the whole brain. Hippocampal tissues were suspended in a sample buffer [29], which consisted of 30 mM Tris, 7 M urea, 2 M thiourea, 65 mM dithiothreitol (DTT), 4% 3-[(3-cholamidopropyl)dimethylammonio]-1-propanesulfonate (CHAPS) with 40 μL protease inhibitor (pH 8.5). Suspensions were sonicated five times for 10 sec and centrifuged at 45,000 rpm for 45 min. Proteins in the supernatants were quantified using the 2D Quant kit (GE Healthcare, Uppsala, Sweden).

#### 2.5.2. Analysis of 2DE Gels

Processed protein (1 mg) was sequentially electrofocused on immobilized pH gradient strips (pH 3–10 nonlinear) and an Ettan IPGphor (GE Healthcare) with 24 cm immobilized pH gradient (IPG) strips (pH 4–7, GE Healthcare), as previously described [29]. After equilibration of strips, they were transferred to 9%–16% SDS-PAGE on an Ettan DALT 12 system (GE Healthcare) and then a preparative gel was stained with Coomassie brilliant blue G250 dye solution overnight, destained using ultrapure distilled water, and scanned using a GS710 scanning densitometer (Bio-Rad, Hemel Hempstead, UK). The gel images were analyzed with Melanie 7 image analysis software (GE Healthcare). Labeled images were uniformly processed using Adobe Photoshop (version CC2014) software.

#### 2.5.3. Trypsin Digestion

Spots of interest were defined if they were found increased or decreased more than twice. These spots were excised from each gel and transferred into 1.5 mL tubes as previously described [10,29]. Each spot was washed with 100 μL of distilled water; then, 50 μL of a 50 mM NH_4_HCO_3_ (pH 7.8) and acetonitrile (6:4) solution was added and the tube was agitated for 10 min. This process was repeated at least three times until the Coomassie brilliant blue G250 dye disappeared. The supernatant was decanted, and the spots were dried in a speed vacuum concentrator (LaBoGeneAps, Lynge, Denmark) for 10 min. Then, 100 ng per spot was digested with trypsin (Promega, Southampton, UK) in 50 mM ammonium bicarbonate and left on ice for 45 min. Spots were then incubated at 37 °C for 12 h.

#### 2.5.4. Protein Identification Using MALDI-TOF MS

Tryptic peptides were desalted and purified using a mixture of Poros R2 and Oligo R3 (Applied Biosystems, Foster City, CA). The MS spectra of peptides were generated by spectrometric analysis using a 4800 MALDI-TOF analyzer (Applied Biosystems) in the reflectron/delayed extraction mode, with an accelerating voltage of 20 kV. Data were summed from 500 laser pulses. The operating software used was Applied Biosystems 4000 series Data Explorer version 4.4 and the T2D file that was obtained from the 4800 MALDI-TOF was opened in Data Explorer. The peaks were filtered using the following four macro processes: (1) baseline correction (peak width = 32, flexibility = 0.5, degree = 0.1), (2) noise filter/smooth: filter coefficient = 0.7, (3) spectrum peak deisotoping: adduct = H, generic formula = C_6_H_5_NO, and (4) mass calibration. The spectrum was calibrated with the reference to tryptic autodigested peaks [m/z 842.5090, 1045.564, and 2211.1046] and monoisotopic peptide masses were obtained with Data Explorer. An 800–4000 m/z mass range was used with 1000 shots per spectrum. At the end of the macro process, raw data were generated about centroid mass, resolution, height, and S/N ratio of each peak. These data were converted into an Excel file and entered into a MASCOT search.

#### 2.5.5. Data Searches for Protein Identification

MASCOT (Matrix Science, London, U.K.; version 2.2.04) was used to identify peptide sequences present in the protein sequence database NCBInr (Mouse). The tryptic peptide masses were used to search for protein candidates using the web-based searching software ProFound.

### 2.6. Validation by Western Blot Analyses

To confirm the differentially expressed proteins in the hippocampus, mice (*n* = 6 in each group) in control and Pyr-def groups were anesthetized with a mixture of alfaxalone and xylazine and hippocampal tissues were obtained from the brain, as previously described [10]. In brief, 500 μm thick sections of each brain were generated using a vibratome (Leica Microsystems, GmbH, Germany), and the hippocampal area was dissected using a surgical blade. The tissues were homogenized in 50 mM PBS (pH 7.4), containing 0.1 mM ethylene glycol-bis(2-aminoethylether)-N,N,N′,N′-tetraacetic acid (pH 8.0); 0.2% Nonidet P-40; 10 mM ethylenediaminetetraacetic acid (pH 8.0); 15 mM sodium pyrophosphate; 100 mM β-glycerophosphate; 50 mM NaF; 150 mM NaCl; 2 mM sodium orthovanadate; 1 mM phenylmethylsulfonyl fluoride; and 1 mM DTT. After centrifugation, protein levels in the supernatants were determined using a Micro BCA protein assay kit according to the manufacturer’s instructions (Pierce Chemical, Rockford, IL, USA). Aliquots containing 20 μg of total protein were denatured by boiling in loading buffer containing 150 mM Tris (pH 6.8), 3 mM DTT, 6% sodium dodecyl sulfate, 0.3% bromophenol blue, and 30% glycerol. Each aliquot was loaded onto a polyacrylamide gel. After electrophoresis, proteins were transferred to nitrocellulose membranes (Pall Crop, East Hills, NY, USA), which were then blocked in 5% non-fat dry milk in PBS/0.1% Tween 20 for 45 min, prior to incubation with a rabbit anti-ATP6V1B2 antibody (diluted 1:500, Abcam, Cambridge, UK) and a rabbit anti-HSC70 antibody (1:200, Abcam). Detection was performed using the peroxidase-conjugated IgG and an enhanced luminol-based chemiluminescent kit (Pierce Chemical). The blots were scanned, and densitometry was performed, using Scion Image software (Scion Corp., Frederick, MD, USA). Blots were stripped and re-probed with an antibody against β-actin as an internal loading control. Data were normalized to the β-actin levels in each lane.

### 2.7. Data Analysis

Five tissue sections per antibody between 1.46 mm and 2.46 mm posterior to the bregma were examined using an image analysis system and ImageJ software v. 1.5 (National Institutes of Health, Bethesda, MD, USA). Digital images of the mid-point of the hippocampal dentate gyrus were captured with a BX51 light microscope (Olympus, Tokyo, Japan) equipped with a digital camera (DP72, Olympus) connected to a computer monitor. Images were calibrated into an array of 512 × 512 pixels corresponding to a tissue area of 1200 μm × 900 μm (100× primary magnification). Each pixel resolution was 256 gray levels; the intensity of DCX immunoreactivity were evaluated by relative optical density (ROD), which was obtained after transformation of the mean gray level using the formula: ROD = log(256/mean gray level). The ROD of background staining was determined in unlabeled portions of the sections using Photoshop CC software (Adobe Systems Inc., San Jose, CA, USA), and this value was subtracted to correct for nonspecific staining, using ImageJ v. 1.50 software (National Institutes of Health). Data are expressed as a percentage of the vehicle-treated group values (set to 100%).

Ki67-positive cell counts were performed for each section of the dentate gyrus using an image analysis system equipped with a computer-based CCD camera (software: Optimas 6.5, CyberMetrics, Scottsdale, AZ, USA). Cell counts from all the sections (*n* = 5) of all of the mice (*n* = 7) were averaged.

### 2.8. Statistical Analysis

Data represent the mean ± the standard error of the mean (SEM). Differences among means were statistically analyzed using the two-way analysis of variance (ANOVA) test, followed by Bonferroni post-hoc tests on novel object recognition, and a Student’s *t*-test to test statistical significance in monoamine changes, proliferating cells, and differentiated neuroblasts in mice. Statistical significance was considered at *p* < 0.05.

## 3. Results

### 3.1. Effects of Pyridoxine Deficiency on PLP Levels in Serum and Brain Tissues

**Figure 1:**
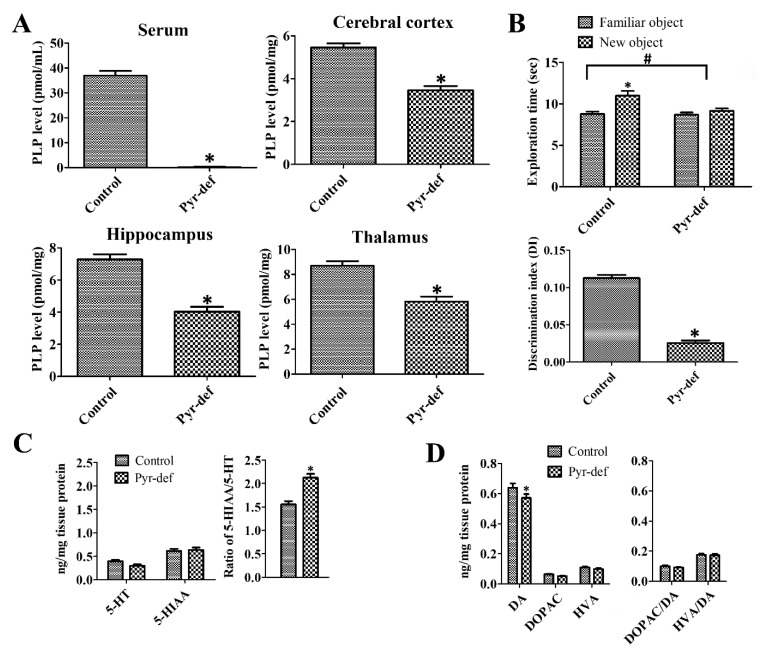
Pyridoxine deficiency (Pyr-def) decreases serum and brain pyridoxal 5′-phosphate (PLP) levels, novel object recognition memory, and monoamine levels in the hippocampus. (**A**) PLP concentration in serum and brain regions (cerebral cortex, hippocampus, and thalamus); data are analyzed with the Student’s *t*-test (*n* = 20 per group; * *p* < 0.05). (**B**) Exploration time for familiar and new objects and the discrimination index in control and Pyr-def mice; data were analyzed with two-way ANOVA followed by Bonferroni post-tests (*n* = 7 per group; * *p* < 0.05, significant difference between familiar and new object, # *p* < 0.05, significant difference between control and Pyr-def group) and Student’s *t*-test (*n* = 7 per group; * *p* < 0.05). (**C**) 5-Hydroxytryptamine (5-HT), its metabolite (5-HIAA), and ratio (5-HIAA/5-HT) in the hippocampus of control and Pyr-def mice measured with HPLC analysis (*n* = 7 mice per group; * *p* < 0.05). (**D**) Levels of 3,4-Dihydroxyphenethylamine (DA), 3,4-dihydroxyphenylacetic acid (DOPAC), homovanillic acid (HVA), and ratio (DOPAC/DA and HVA/DA) in the hippocampus of control and Pyr-def mice measured through HPLC analysis (*n* = 7 mice per group; * *p* < 0.05).

In the control group, the PLP levels were 36.9 ± 1.91 pmol/mL in the serum and 5.46 ± 0.19, 7.28 ± 0.32, 8.69 ± 0.99 pmol/mg in the cerebral cortex, hippocampus, and thalamus, respectively. In the Pyr-def group, PLP levels were dramatically decreased compared to those in the control group and were nearly undetectable. PLP levels in the cerebral cortex, hippocampus, and thalamus homogenates significantly decreased to 59.9%, 55.4%, and 67.0% of the control group levels, respectively (Figure 1A).

### 3.2. Effects of Pyridoxine Deficiency on Novel Object Recognition

There are no significant differences in the total exploratory activity in both control and Pyr-def groups at the training and test trials of the novel object recognition test although the exploratory behavior slightly decreased in Pyr-def group (Supplementary Figure S1). However, the exploration time to familiar and new objects showed prominent differences between groups. At the training trial, mice in both groups spent a similar amount of time exploring two identical objects. At the testing trial, control mice spent more time exploring the new object (10.998 ± 0.559 sec) than the familiar one (8.765 ± 0.298 sec), while in the Pyr-def group, mice spent a similar time exploring the new (9.153 ± 0.319 sec) and familiar (8.694 ± 0.275 sec) objects. Two-way ANOVA testing revealed that pyridoxine deficiency (F = 12.52, DFn = 1, Dfd = 24, *p* = 0.017) as well as familiar and novel objects (F = 6.34, DFn = 1, Dfd = 24, *p* = 0.0189) demonstrated the significant effects on the results. In addition, pyridoxine deficiency showed the same effects on time to explore the familiar and novel objects (F = 5.438, DFn = 1, Dfd = 24, *p* = 0.0284). The existence of significant changes in exploring time in new and familiar objects of control and Pyr-def groups The DI was significantly lower in the Pyr-def group compared to that in the control group (*p* = 0.0006) (Figure 1B).

### 3.3. Effects of Pyridoxine Deficiency on Monoamine Levels in the Hippocampus

In the control group, 5-HT and 5-HIAA levels in hippocampal homogenates were 0.40 ± 0.026 and 0.61 ± 0.044 ng/mg protein, respectively and the ratio of 5-HIAA/5-HT was 1.53. In the Pyr-def group, the 5-HT level was decreased prominently, but not significantly, while 5-HIAA levels were similar compared to those in the control group. However, the ratio of 5-HIAA/5-HT in the Pyr-def group was significantly higher than that in the control group (Figure 1C).

In the control group, DA, DOPAC, and HVA levels were 0.64 ± 0.030, 0.064 ± 0.0032, and 0.11 ± 0.0065 nmol/mg protein, respectively. Ratios of DOPAC/DA and HVA/DA in the control group were 0.10 and 0.18, respectively. In the Pyr-def group, DA levels were significantly lower in the hippocampal homogenates compared to the control group, and DOPAC, and HVA levels were decreased compared to those in the control group, respectively although no statistical significance was detected between groups. Ratios of DOPAC/DA and HVA/DA in the Pyr-def group were similar to those in the control group (Figure 1D).

### 3.4. Effects of Pyridoxine Deficiency on Ki67 and DCX Immunoreactivity

**Figure 2:**
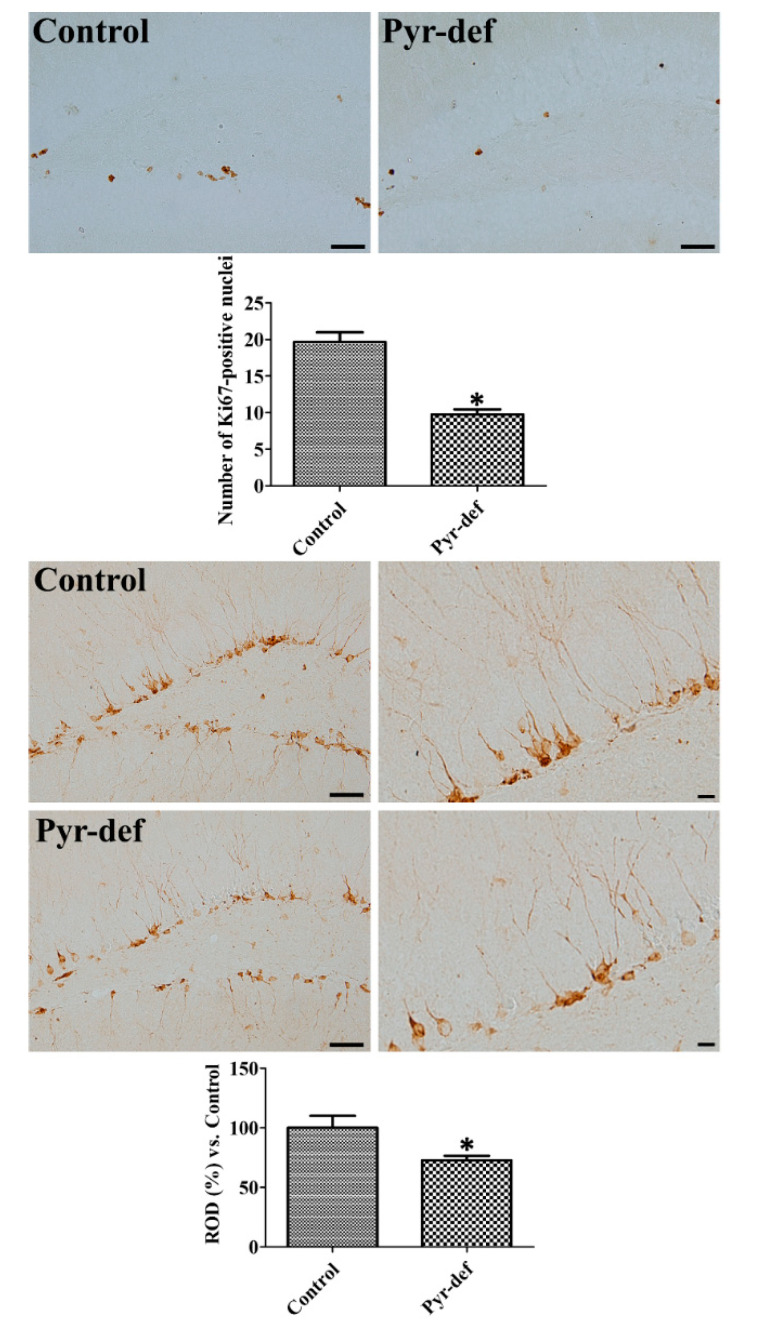
Pyridoxine deficiency (Pyr-def) decreases Ki67-positive proliferating cells and doublecortin- (DCX) immunoreactive neuroblasts in the dentate gyrus. (Upper) Photomicrographs of Ki67-positive structures in the dentate gyrus of control and Pyr-def mice. Scale bar = 50 μm. The number of Ki67-positive nuclei is counted in 5 sections located in every 90-μm intervals (*n* = 7 mice per group; * *p* < 0.05). (Lower) Photomicrographs of DCX-immunoreactive structures in the dentate gyrus of control and Pyr-def mice. Scale bar = 50 μm. Relative optical density (ROD) corresponds to the percentage of DCX immunoreactivity value in the dentate gyrus of the control group (*n* = 7 mice per group; * *p* < 0.05). All data are expressed as mean ± SEM.

In the control and Pyr-def groups, Ki67-positive proliferating cells were found in the subgranular zone of the dentate gyrus. However, the number of Ki67-positive cells in the Pyr-def group was 49.6% (*p* = 0.0006) of that in the control group (Figure 2).

In the control and Pyr-def groups, cell bodies of DCX immunoreactive neuroblasts were found in the subgranular zone of the dentate gyrus and their dendrites extended into the molecular layer of dentate gyrus. In the Pyr-def group, DCX immunoreactive neuroblasts and their dendrites were less abundantly observed, and DCX immunoreactivity was significantly decreased to 72.8% (*p* = 0.0348) of that in the control group (Figure 2).

### 3.5. Protein Identification by 2D-DIGE Followed by MALDI-TOF MS in the Hippocampus

**Figure 3:**
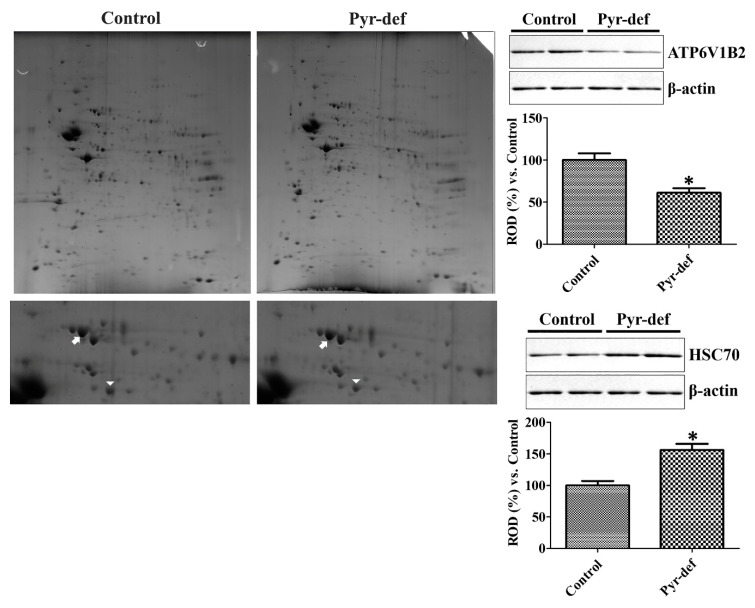
Two-dimensional gel electrophoresis (2DE) and subsequent matrix-assisted laser desorption/ionization time-of-flight mass spectrometry (MALDI-TOF MS) analysis of the hippocampus of control and pyridoxine deficient diet-fed mice. (Left) 2DE gels are enlarged and each panel shows an enlarged view of 2DE gel spots that were expressed differentially (arrowheads and arrows are ATP6V1B2 and HSC70, respectively). (Right) Western blot analysis of differentially expressed proteins (ATP6V1B2 and HSC70) in the hippocampus of control and Pyr-def mice (*n* = 6 per group; * *p* < 0.05, vs. vehicle-treated group). All data are shown as the ratio to control group ± SEM.

In control and Pyr-def groups, 449 and 468 of total spots were detected, respectively, in the in-gel module and 378 spots were paired. Among these 378 paired spots, a total of 20 spots increased more than two-fold, while 21 spots were decreased (Figure 3).

Based on SWISS-PROT and NCBInr databases, 10 spots showed no significant hits to report because of small amounts of the peptide. The other 31 spots were identified based on MALDI-TOF MS analysis and are listed in Table 1 and Table 2. Among these identified candidate proteins, we considered several factors such as the number of matched peptides, coverage, and the theoretical MOlecular Weight Search (MOWSE) score. The most reliable proteins, which had a high number of peptides matched in the identified protein and protein sequence coverage, were V-type proton ATPase subunit B, brain isoform (ATP6V1B2) and heat shock cognate protein 70 (HSC70). The standard abundance values of ATP6V1B2 were significantly lower, while those of HSC70 were significantly higher in the Pyr-def group compared to the control (Figure 3).

### 3.6. Validation of Identified Proteins by Western Blot

The expression of ATP6V1B2 and HSC70 was validated by Western blot analysis. ATP6V1B2 expression was significantly decreased in the hippocampal homogenates of the Pyr-def group by 61.2% of that in the control group. In contrast, HSC70 protein levels in the Pyr-def group were significantly increased to 156.3% of those in the control group (Figure 3).

## 4. Discussion

Pyridoxine is one of the essential elements during brain development and acts as a cofactor in synthesizing 5-HT, norepinephrine, DA, melatonin, and other neurotransmitters. Several lines of evidence demonstrate abnormalities in locomotor and learning behavior in offspring when maternal pyridoxine deficiency is induced [30,31,32]. Maternal pyridoxine deficiency significantly reduces serotonergic [33] and DAergic activity [34,35] as well as increases plasma homocysteine levels by reducing cystathionine synthase activity in pups [36,37]. In contrast, adult animals show relative resistance to pyridoxine deficiency in adulthood [38], although vitamin B6 deficiency affects T-lymphocyte cell numbers, decreases IL-2, and increases IL-4 in spleen lymphocytes [39]. In the present study, we focused upon the effects of pyridoxine deficiency on hippocampal functions during the juvenile and young adult periods. Pyr-def diet significantly reduced serum PLP levels to nearly undetectable levels in all animals and PLP levels were significantly decreased in the cerebral cortex, hippocampus, and thalamus in Pyr-def diet-fed mice. These results showed that the Pyr-def animal model was successfully established with consistent results with those of previous studies [39]. 

In the present study, we observed the effects of pyridoxine deficiency on novel object recognition memory to assess the hippocampal function [40]. Pyridoxine deficiency significantly reduced the novel object recognition memory, based on the difference in the discrimination index between the Pyr-def and control groups. An electron microscopy study shows axonal swellings in vitamin B_6_-deficient rats [41]. Moreover, dietary deficiency of folate, vitamin B_6_, and B_12_ significantly increases Aβ deposits in the hippocampus [42]. In line with these reports, pyridoxine improves novel object recognition memory in healthy mice [10] and spatial memory in isocarbophos-induced vascular dementia rats tested on the Morris water maze [43]. In addition, *PLP phosphatase* knockout mice exhibit improved spatial learning and memory compared to controls [22]. Although a previous study showed that maternal pyridoxine deficiency affects hippocampal electrical activity in offspring [24], our study provides new insight into memory impairment induced by pyridoxine deficiency in juvenile and young-adult mice.

Maternal pyridoxine deficiency decreases serotonergic [33] and DAergic [34,35] signaling. In this study, we investigated the effects of pyridoxine deficiency in 5-HT, DA, and metabolite levels in adulthood. We observed the reduction of 5-HT levels in the hippocampus, although no statistical significance was detected between control and Pyr-def fed groups. In addition, 5-HIAA levels showed similar content in both groups. This result suggests that pyridoxine deficiency decreased the 5-HT levels, not its metabolism, in the hippocampus. Constitutive depletion of 5-HT results in deficits in novel object recognition memory [44] and targeting of the serotonergic terminal in the hippocampus impairs spatial memory [45]. In contrast, activation of the serotonergic terminal in the hippocampus improves spatial memory [45]. We also observed that pyridoxine deficiency resulted in a significant reduction of DA levels in the hippocampus compared to the control group, although the levels of the metabolites DOPAC and HVA did not demonstrate any significant changes between groups. DA innervations of the hippocampus from locus coeruleus are involved in spatial learning and memory [46], while the depletion of DA significantly decreases the novel object recognition memory in mice [47]. 

Next, we investigated the effects of pyridoxine deficiency on proliferating cells and neuroblasts in the dentate gyrus because the recognition memory is closely related to hippocampal neurogenesis [48]. Pyridoxine deficiency significantly reduced the Ki67-positive proliferating cells and DCX-immunoreactive neuroblasts compared to those in the control group. Maternal pyridoxine deficiency decreases the total number of neurons in the neocortex and reduces the dendrites in postnatal rat pups [49]. In agreement with this, we previously demonstrated that pyridoxine treatment significantly increases proliferating cells and differentiated neuroblasts in the dentate gyrus [10,12]. Collectively, these results suggest that pyridoxine is an essential element of hippocampal neurogenesis.

To identify the possible regulatory proteins of pyridoxine deficiency in the hippocampus, we conducted 2-DE and subsequent MALDI-TOF analysis. We found ATP6V1B2 and HSC70 as candidate proteins involved in pyridoxine regulation based on high coverage and MOWSE scores. Validation by Western blot analysis showed that pyridoxine deficiency significantly decreased ATP6V1B2 expression in the hippocampus, while HSC70 was significantly increased in the Pyr-def group compared to that in the control group. ATP6V1B2 is ubiquitously expressed in the brain [50] and is related to synaptic transmission [51]. *Atp6v1b2* knock-in mice show cognitive defects in passive avoidance and novel object recognition tests [52] and the ATP6V1B2 rs1106634 A allele is a risk factor in hippocampal cognitive deficits [53]. HSC70 is upregulated in Alzheimer’s disease brains [54]. An HSC70 inhibitor improves the novel object recognition memory in Alzheimer’s disease mice [55] and decreases the levels of the tau protein [55,56,57,58].

In conclusion, pyridoxine deficiency affects the novel object recognition memory and hippocampal neurogenesis, which are associated with the decrease of ATP6V1B2 and increase of HSC70 levels in the hippocampus. Collectively, pyridoxine is an essential element in neurogenesis and hippocampal cognitive functions.

## Figures and Tables

**Table 1:** Identified candidate proteins in hippocampal homogenates decreased after pyridoxine-deficient diet treatment more than two-folds. Note that only V-type proton ATPase subunit B2 (ATP6V1B2) shows more than 20 peptides matched in the identified protein and protein sequence coverage.

Proteins (gI Accession Number)	Number of Peptides Matched in the Identified Protein	Protein Sequence Coverage (%)	pI, Mr (kDa)	MOWSE	Fold Decrease
APG-1 (705391)	1	1	5.53, 95.3	39	
Zinc finger protein 831 (150010581)	1	0	8.18 176.1	40	2.3
Murine valosin-containing protein (55217)	3	3	5.14 89.9	105	2.5
Heat-shock protein hsp84 (194027)	2	3	4.95 83.5	89	2.0
V-type proton ATPase subunit B2 (17105370)	38	25	5.57 56.9	638	2.3
Vacuolar adenosine triphosphatase subunit B (1184661)	1	2	5.57 56.9	45	3.1
Acyltransferase (1129118)	1	2	8.88 53.5	48	2.8
Anti-DNA immunoglobulin light chain IgG (1870366)	1	8	9.11 11.1	39	2.1
Poly(rC)-binding protein 1 (6754994)	1	2	6.66 38.0	40	2.2
Guanine nucleotide-binding protein G(I)/G(S)/G(T) subunit beta-1 (6680045)	12	12	5.60 38.2	243	8.8
Glyceraldehyde-3-phosphate dehydrogenase (2494630)	1	1	8.14 48.4	34	3.0
Charged multivesicular body protein 4b (2807749)	2	11	4.76 24.9	85	2.1
Voltage-dependent anion-selective channel protein 2 (6755965)	3	7	7.44 32.3	102	3.4
Serine-threonine kinase receptor-associated protein (4063383)	1	3	4.99 38.8	36	2.8
Osmotic stress protein 94 (1098541)	5	5	5.50 95.2	215	3.3

**Table 2:** Identified candidate proteins in hippocampal homogenates increased after pyridoxine-deficient diet treatment more than two-folds. Note that heat shock cognate protein 70 (HSC70) and heat shock-related 70 kDa protein 2 shows more than 20 peptides matched in the identified protein and protein sequence coverage.

Proteins (gI Accession Number)	Number of Peptides Matched in the Identified Protein	Protein Sequence Coverage (%)	pI, Mr (kDa)	MOWSE	Fold Increase
NADH dehydrogenase (13879366)	2	2	5.51 80.7	68	2.0
Heat shock cognate protein 70 (309319)	49	29	5.37 71.0	1066	2.4
Heat shock-related 70 kDa protein 2 (31560686)	20	21	5.51 69.9	620	3.5
Dihydrolipoamide dehydrogenase (2078522)	1	2	7.97 54.7	41	2.3
ATP synthase subunit alpha, mitochondrial precursor (6680748)	10	9	9.22 59.8	308	2.7
Aldehyde dehydrogenase, mitochondrial isoform 1 precursor (6753036)	2	3	7.53 57.0	96	2.0
Type II keratin subunit protein (4159806)	1	1	8.97 65.7	40	2.8
Spermatogenesis-associated protein 7 homolog isoform 1	1	1	6.23 66.2	34	3.0
ATP-specific succinyl-CoA synthetase beta subunit	16	13	5.65 46.6	366	3.6
Protein phosphatase type 2A catalytic subunit alpha isoform (3342500)	1	2	5.30 36.2	48	2.4
Histidine triad nucleotide-binding protein 1 (33468857)	1	11	6.36 13.9	41	2.1
Epidermal keratin subunit I, partial (387397)	17	8	5.01 58.0	304	4.0
2-Oxoglutarate dehydrogenase-like, mitochondrial (568987556)	2		83.2	96	2.4

## Supplementary Materials

The following are available online at https://www.mdpi.com/2073-4409/9/5/1067/s1, Figure S1: Pyridoxine deficiency has no effects on total exploratory activity in control and Pyr-def mice; data were analyzed with two-way ANOVA followed by Bonferroni post-tests.
