# Supplementary material for: Effects of Pyridoxine Deficiency on Hippocampal Function and Its Possible Association with V-Type Proton ATPase Subunit B2 and Heat Shock Cognate Protein 70
Source: Cells. 2020 Apr 25;9(5):1067. doi: 10.3390/cells9051067 (PMC7290376; doi:10.3390/cells9051067)
Supplement: Supplementary file 1 [file cells-09-01067-s001.pdf]

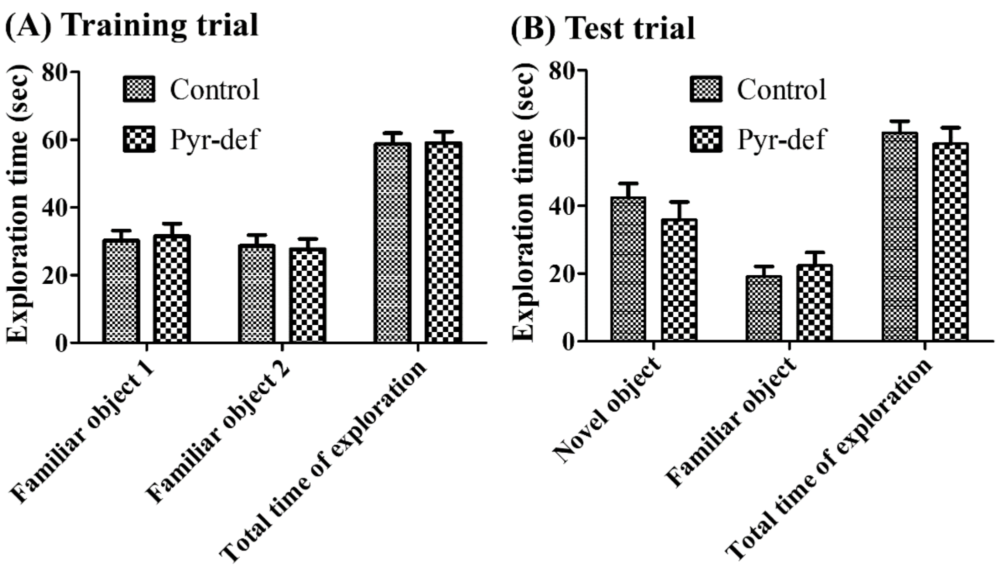

Supplementary Figure 1. Pyridoxine deficiency has no effects on total exploratory activity in control and Pyr-def mice; data were analyzed with two-way ANOVA followed by Bonferroni post-tests (n = 7 per group).

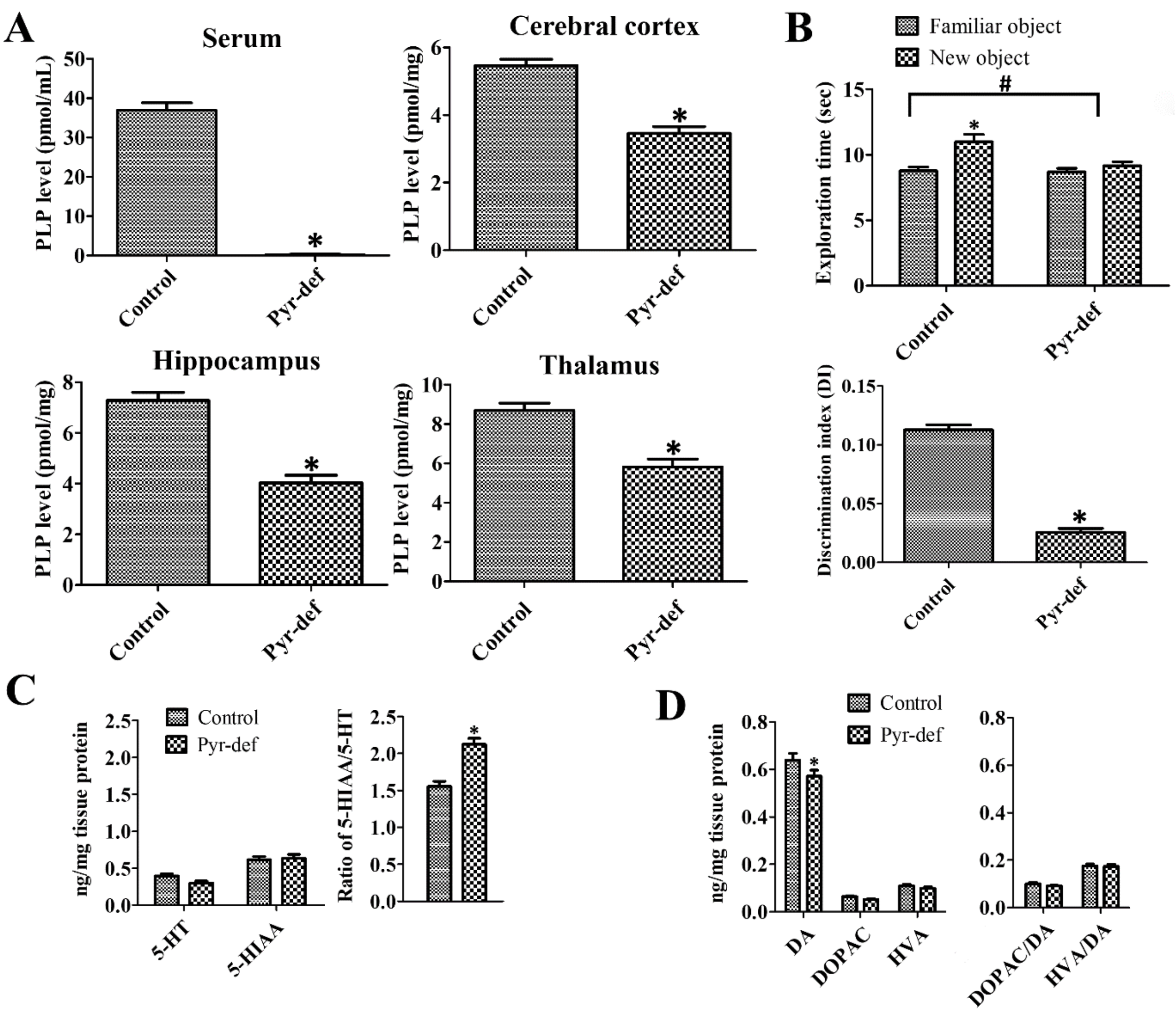

Fig. 1. Pyridoxine deficiency (Pyr-def) decreases serum and brain PLP levels, novel object recognition memory, and monoamine levels in the hippocampus. (A) PLP concentration in serum and brain regions (cerebral cortex, hippocampus and thalamus); data are analyzed with the Student's t-test (n = 20 per group; *$p < 0.05$). (B) Exploration time for familiar and new objects and the discrimination index in control and Pyr-def mice; data were analyzed with two-way ANOVA followed by Bonferroni post-tests (n = 7 per group; *$p < 0.05$, significant difference between familiar and new object, #$p < 0.05$, significant difference between control and pyr-def group) and Student's t-test (n = 7 per group; *$p < 0.05$). (C) 5-Hydroxytryptamine (5-HT), its metabolite (5-HIAA), and ratio (5-HIAA/5-HT) in the hippocampus of control and Pyr-def mice measured with HPLC analysis (n = 7 mice per group; *$P < 0.05$). (D) Levels of 3,4-Dihydroxyphenethylamine (DA), 3,4-dihydroxyphenylacetic acid (DOPAC), homovanillic acid (HVA), and ratio (DOPAC/DA and HVA/DA) in the hippocampus of control and Pyr-def mice measured through HPLC analysis (n = 7 mice per group; *$P < 0.05$).

3.4. *Effects of pyridoxine deficiency on Ki67 and DCX immunoreactivity*

In the control and Pyr-def groups, Ki67-positive proliferating cells were found in the subgranular zone of the dentate gyrus. However, the number of Ki67-positive cells in the Pyr-def group was 49.6% (p = 0.0006) of that in the control group (Fig. 2A).

In the control and Pyr-def groups, cell bodies of DCX immunoreactive neuroblasts were found in the subgranular zone of dentate gyrus and their dendrites extended into the molecular layer of dentate gyrus. In the Pyr-def group, DCX immunoreactive neuroblasts and their dendrites were less abundantly observed and DCX immunoreactivity was significantly decreased to 72.8% (p = 0.0348) of that in the control group (Fig. 2B).

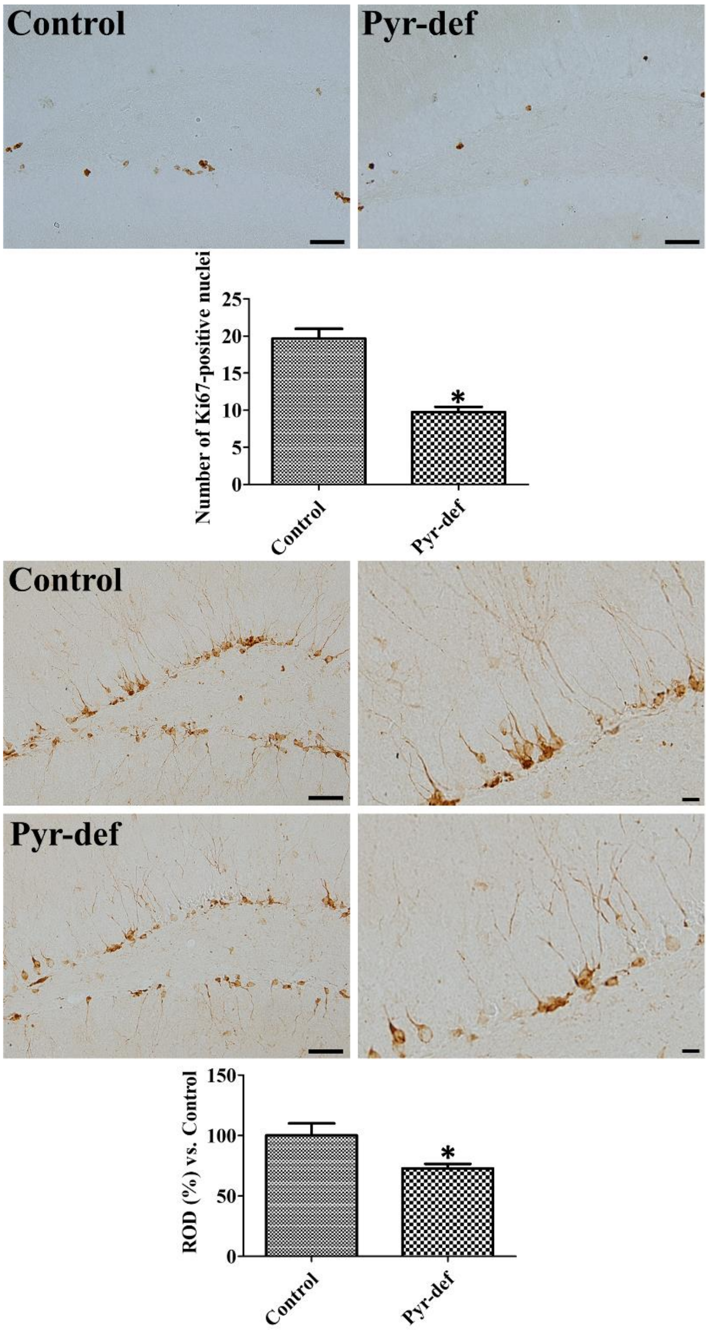

Fig. 2. Pyridoxine deficiency (Pyr-def) decreases Ki67-positive proliferating cells and doublecortin-(DCX) immunoreactive neuroblasts in the dentate gyrus. (Upper) Photomicrographs of Ki67-positive structures in the dentate gyrus of control and Pyr-def mice. Scale bar = 50 μm. The number of Ki67-positive nuclei is counted in 5 sections located in every 90-μm intervals (n = 7 mice per group; *P < 0.05). (Lower) Photomicrographs of DCX-immunoreactive structures in the dentate gyrus of control and Pyr-def mice. Scale bar = 50 μm. Relative optical density (ROD) corresponds to the percentage of DCX

immunoreactivity value in the dentate gyrus of the control group (n = 7 mice per group; *$P < 0.05$). All data are expressed as mean ± SEM.

3.5. Protein identification by 2D-DIGE followed by MALDI-TOF MS in the hippocampus

In control and Pyr-def groups, 449 and 468 of total spots were detected, respectively, in the in-gel module and 378 spots were paired. Among these 378 paired spots, a total of 20 spots increased more than two-fold, while 21 spots were decreased (Fig. 3A).

Based on SWISS-PROT and NCBInr databases, 10 spots showed no significant hits to report because of small amounts of peptide. The other 31 spots were identified based on MALDI-TOF MS analysis and are listed in Tables 1 and 2. Among these identified candidate proteins, we considered several factors such as the number of matched peptides, coverage, and the theoretical MOlecular Weight Search (MOWSE) score. The most reliable proteins, which had high number of peptides matched in the identified protein and protein sequence coverage, were V-type proton ATPase subunit B, brain isoform (ATP6V1B2) and heat shock cognate protein 70 (HSC70). The standard abundance values of ATP6V1B2 were significantly lower, while those of HSC70 were significantly higher in the Pyr-def group compared to the control (Fig. 3A).

3.6. Validation of identified proteins by Western blot

The expression of ATP6V1B2 and HSC70 was validated by Western blot analysis. ATP6V1B2 expression was significantly decreased in the hippocampal homogenates of the Pyr-def group by 61.2% of that in the control group. In contrast, HSC70 protein levels in the Pyr-def group were significantly increased to 156.3% of those in the control group (Fig. 3B).

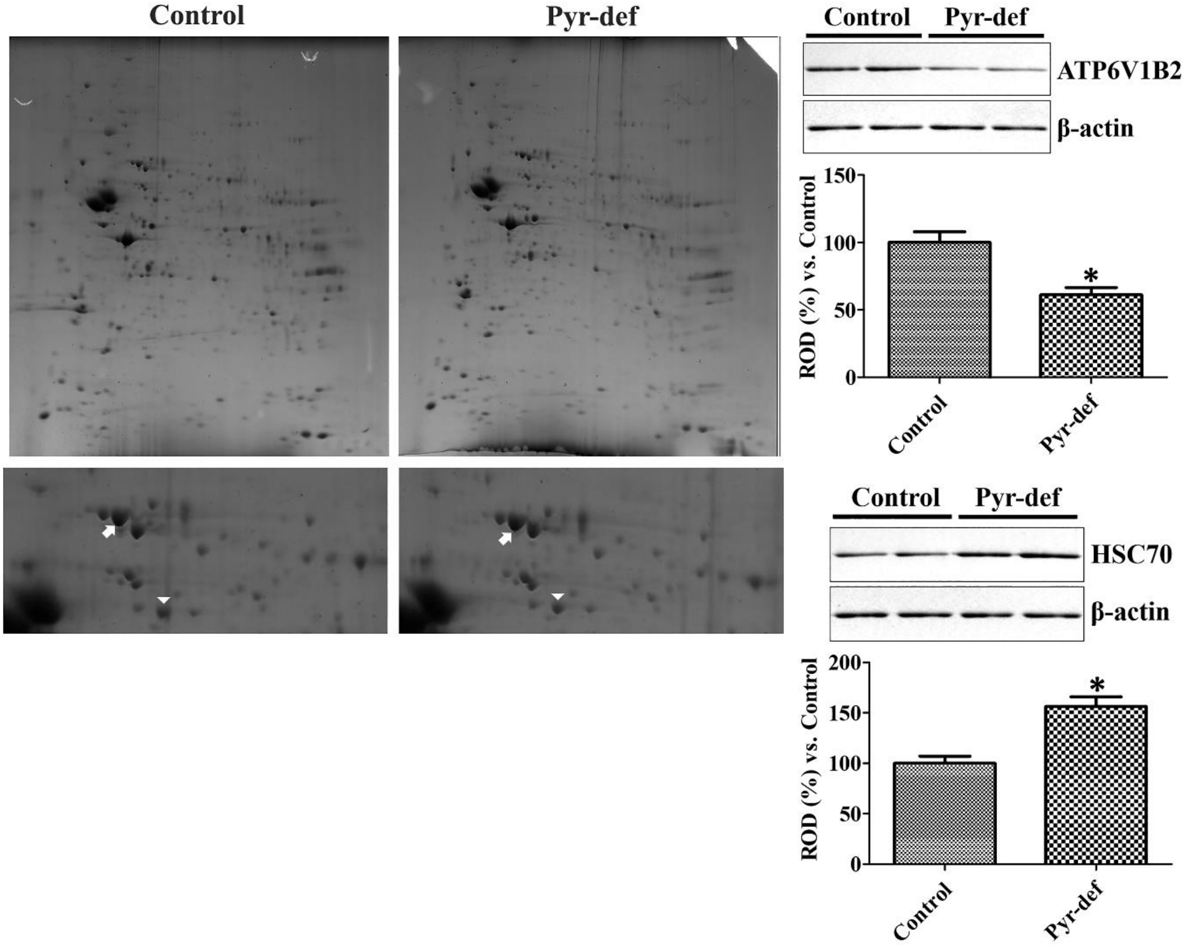

Fig. 3. Two dimensional gel electrophoresis (2DE) and subsequent matrix-assisted laser desorption/ionization time-of-flight mass spectrometry (MALDI-TOF MS) analysis of the hippocampus of control and pyridoxine deficient diet fed mice. (Left) 2DE gels are enlarged and each panel shows an enlarged view of 2DE gel spots that were expressed differentially (arrowheads and arrows are ATP6V1B2 and HSC70, respectively). (Right) Western blot analysis of differentially expressed proteins (ATP6V1B2 and HSC70) in the hippocampus of control and Pyr-def mice (*n* = 6 per group; *$P < 0.05$, vs. vehicle-treated group). All data are shown as the ratio to control group ± SEM.

Table 1. Identified candidate proteins in hippocampal homogenates decreased after pyridoxine-deficient diet treatment more than two-folds. Note that only V-type proton ATPase subunit B2 (ATP6V1B2) shows more than 20 peptides matched in the identified protein and protein sequence coverage.

**Proteins (gI accession number)**	**Number of peptides matched in the identified protein**	**Protein sequence coverage (%)**	**pI, Mr (kDa)**	**MOWSE**	**Fold decrease**
APG-1 (705391)	1	1	5.53, 95.3	39	
Zinc finger protein 831	1	0	8.18 176.1	40	2.3

(150010581)					
Murine valosin-containing protein (55217)	3	3	5.14<br>89.9	105	2.5
Heat-shock protein hsp84 (194027)	2	3	4.95<br>83.5	89	2.0
V-type proton ATPase subunit B2 (17105370)	38	25	5.57<br>56.9	638	2.3
Vacuolar adenosine triphosphatase subunit B (1184661)	1	2	5.57<br>56.9	45	3.1
Acyltransferase (1129118)	1	2	8.88<br>53.5	48	2.8
Anti-DNA immunoglobulin light chain IgG (1870366)	1	8	9.11<br>11.1	39	2.1
Poly(rC)-binding protein 1 (6754994)	1	2	6.66<br>38.0	40	2.2
Guanine nucleotide-binding protein G(I)/G(S)/G(T) subunit beta-1 (6680045)	12	12	5.60<br>38.2	243	8.8
Glyceraldehyde-3-phosphate dehydrogenase	1	1	8.14<br>48.4	34	3.0

(2494630)					
Charged multivesicular body protein 4b (2807749)	2	11	4.76 24.9	85	2.1
Voltage-dependent anion-selective channel protein 2 (6755965)	3	7	7.44 32.3	102	3.4
Serine-threonine kinase receptor-associated protein (4063383)	1	3	4.99 38.8	36	2.8
Osmotic stress protein 94 (1098541)	5	5	5.50 95.2	215	3.3

Table 2. Identified candidate proteins in hippocampal homogenates increased after pyridoxine-deficient diet treatment more than two-folds. Note that heat shock cognate protein 70 (HSC70) and heat shock-related 70 kDa protein 2 shows more than 20 peptides matched in the identified protein and protein sequence coverage.

**Proteins (gI accession number)**	**Number of peptides matched in the identified protein**	**Protein sequence coverage (%)**	**pI, Mr (kDa)**	**MOWSE**	**Fold increase**
NADH dehydrogenase (13879366)	2	2	5.51 80.7	68	2.0
Heat shock cognate protein 70 (309319)	49	29	5.37 71.0	1066	2.4

Heat shock-related 70 kDa protein 2 (31560686)	20	21	5.51 69.9	620	3.5
Dihydrolipoamide dehydrogenase (2078522)	1	2	7.97 54.7	41	2.3
ATP synthase subunit alpha, mitochondrial precursor (6680748)	10	9	9.22 59.8	308	2.7
Aldehyde dehydrogenase, mitochondrial isoform 1 precursor (6753036)	2	3	7.53 57.0	96	2.0
Type II keratin subunit protein (4159806)	1	1	8.97 65.7	40	2.8
Spermatogenesis-associated protein 7 homolog isoform 1	1	1	6.23 66.2	34	3.0
ATP-specific succinyl-CoA synthetase beta subunit	16	13	5.65 46.6	366	3.6
Protein phosphatase type 2A catalytic subunit alpha isoform (3342500)	1	2	5.30 36.2	48	2.4
Histidine triad nucleotide-binding protein 1	1	11	6.36 13.9	41	2.1

(33468857)					
Epidermal keratin subunit I, partial (387397)	17	8	5.01 58.0	304	4.0
2-Oxoglutarate dehydrogenase-like, mitochondrial (568987556)	2		83.2	96	2.4

4. Discussion

Pyridoxine is one of the essential elements during brain development and acts as a cofactor in synthesizing 5-HT, norepinephrine, DA, melatonin, and other neurotransmitters. Several lines of evidence demonstrate abnormalities in locomotor and learning behavior in offspring when maternal pyridoxine deficiency is induced [30-32]. Maternal pyridoxine deficiency significantly reduces serotonergic [33] and DAergic activity [34, 35] as well as increases plasma homocysteine levels by reducing cystathionine synthase activity in pups [36, 37]. In contrast, adult animals show relative resistance to pyridoxine deficiency in adulthood [38], although vitamin B6 deficiency affects T-lymphocyte cell numbers, decreases IL-2, and increases IL-4 in spleen lymphocytes [39]. In the present study, we focused upon the effects of pyridoxine deficiency on hippocampal functions during the juvenile and young adult periods. Pyr-def diet significantly reduced serum PLP levels to nearly undetectable levels in all animals and PLP levels were significantly decreased in the cerebral cortex, hippocampus, and thalamus in Pyr-def diet fed mice. This results showed that Pyr-def animal model was successfully established with consistent results with those of previous studies [39].

In the present study, we observed the effects of pyridoxine deficiency on novel object recognition memory to assess the hippocampal function [40]. Pyridoxine deficiency significantly reduced the novel object recognition memory, based on the difference in the discrimination index between the Pyr-def and control groups. An electron microscopy study shows axonal swellings in vitamin B_6-deficient rats [41]. Moreover, dietary deficiency of folate, vitamin B_6, and B_{12} significantly increases Aβ deposits in the hippocampus [42]. In line with these reports, pyridoxine improves novel object recognition memory in healthy mice [10] and spatial memory in isocarbophos-induced vascular dementia rats tested on the Morris water maze [43]. In addition, *PLP phosphatase* knockout

mice exhibit improved spatial learning and memory compared to controls [22]. Although a previous study showed that maternal pyridoxine deficiency affects hippocampal electrical activity in offspring [24], our study provides new insight into memory impairment induced by pyridoxine deficiency in juvenile and young-adult mice.

Maternal pyridoxine deficiency decreases serotonergic [33] and DAergic [34, 35] signaling. In this study, we investigated the effects of pyridoxine deficiency in 5-HT, DA, and their metabolite levels in adulthood. We observed the reduction of 5-HT levels in the hippocampus, although no statistical significance was detected between control and Pyr-def fed groups. In addition, 5-HIAA levels showed similar content in both groups. This result suggests that pyridoxine deficiency decreased the 5-HT levels, not its metabolism, in the hippocampus. Constitutive depletion of 5-HT results in deficits in novel object recognition memory [44] and targeting of the serotonergic terminal in the hippocampus impairs spatial memory [45]. In contrast, activation of the serotonergic terminal in the hippocampus improves the spatial memory [45]. We also observed that pyridoxine deficiency resulted in significant reduction of DA levels in the hippocampus compared to the control group, although the levels of the metabolites DOPAC and HVA did not demonstrate any significant changes between groups. DA innervations of the hippocampus from locus coeruleus are involved in spatial learning and memory [46], while depletion of DA significantly decreases the novel object recognition memory in mice [47].

Next, we investigated the effects of pyridoxine deficiency on proliferating cells and neuroblasts in the dentate gyrus because the recognition memory is closely related to hippocampal neurogenesis [48]. Pyridoxine deficiency significantly reduced the Ki67-positive proliferating cells and DCX-immunoreactive neuroblasts compared to those in the control group. Maternal pyridoxine deficiency decreases the total number of neurons in the neocortex and reduces the dendrites in postnatal rat pups [49]. In agreement with this, we previously demonstrated that pyridoxine treatment significantly increases proliferating cells and differentiated neuroblasts in the dentate gyrus [10, 12]. Collectively, these results suggest that pyridoxine is an essential element to hippocampal neurogenesis.

To identify the possible regulatory proteins of pyridoxine deficiency in the hippocampus, we conducted 2-DE and subsequent MALDI-TOF analysis. We found ATP6V1B2 and HSC70 as candidate proteins involved in pyridoxine regulation based on high coverage and MOWSE scores. Validation by Western blot analysis showed that pyridoxine deficiency significantly decreased ATP6V1B2 expression in the hippocampus, while HSC70 was significantly increased in the Pyr-def group compared to that in the control group. ATP6V1B2 is ubiquitously expressed in the brain [50] and is related to synaptic transmission [51]. *Atp6v1b2* knock-in mice show cognitive defects in the passive avoidance and novel object recognition tests [52] and the ATP6V1B2 rs1106634 A allele is a risk factor in hippocampal cognitive deficits [53]. HSC70 is upregulated in Alzheimer's disease brains [54]. A

HSC70 inhibitor improves the novel object recognition memory in Alzheimer's disease mice [55] and decreases the levels of the tau protein [55-58].

In conclusion, pyridoxine deficiency affects the novel object recognition memory and hippocampal neurogenesis, which are associated with the decrease of ATP6V1B2 and increase of HSC70 levels in the hippocampus. Collectively, pyridoxine is an essential element in neurogenesis and hippocampal cognitive functions.

Authors' contributions: HYJ, WK, KRH, HJK, SMN, JYC, YSY, DWK, DYY, and IKH conceived the study. HYJ, DYY, and IKH designed the study and wrote the manuscript. HYJ, WK, and KRH conducted the animal experiments. HJK, JYC and DWK conducted biochemical experiments. DYY conducted the behavioral experiment. SMN, JYC, YSY, and DWK participated in designing and discussing the study. All authors have read and approved the final manuscript.

Funding: This work was supported by the Promising-Pioneering Researcher Program through Seoul National University (SNU) in 2015 and by the National Research Foundation of Korea (NRF) grant funded by the Korea government (MSIP) (No. 2019R1A2C1005440). In addition, this study was partially supported by the Research Institute for Veterinary Science of Seoul National University.

Competing interests: The authors declare that they have no competing interests.

References

1. Sibbe, M.; Kulik, A. GABAergic regulation of adult hippocampal neurogenesis. *Mol. Neurobiol.* **2017**, 54, 5497-5510.
2. Hodge, R.D.; Kowalczyk, T.D.; Wolf, S.A.; Encinas, J.M.; Rippey, C.; Enikolopov, G.; Kempermann, G.; Hevner, R.F. Intermediate progenitors in adult hippocampal neurogenesis: Tbr2 expression and coordinate regulation of neuronal output. *J. Neurosci.* **2008**, 28, 3707-3717.
3. Yau, S.Y.; Li, A.; So, K.F. Involvement of adult hippocampal neurogenesis in learning and forgetting. *Neural Plast.* **2015**, 2015, 717958.
4. Freund, T.F.; Buzsáki, G. Interneurons of the hippocampus. *Hippocampus* **1996**, 6, 347-470.
5. Jessberger, S.; Clark, R.E.; Broadbent, N.J.; Clemenson, G.D. Jr.; Consiglio, A.; Lie, D.C.; Squire, L.R.; Gage, F.H. Dentate gyrus-specific knockdown of adult neurogenesis impairs spatial and object recognition memory in adult rats. *Learn. Mem.* **2009**, 16, 147-154.
6. Garrido-Franco, M. Pyridoxine 5'-phosphate synthase: de novo synthesis of vitamin B6 and beyond. *Biochim. Biophys. Acta* **2003**, 1647, 92-97.
7. Lheureux, P.; Penaloza, A.; Gris, M. Pyridoxine in clinical toxicology: a review. *Eur. J. Emerg. Med.* **2005**, 12, 78-85.
8. Mooney, S.; Leuendorf, J.E.; Hendrickson, C.; Hellmann, H. Vitamin B6: a long known compound of surprising complexity. *Molecules* **2009**, 14, 329-351.
9. Baldewicz, T.; Goodkin, K.; Feaster, D.J.; Blaney, N.T.; Kumar, M.; Kumar, A.; Shor-Posner, G.; Baum, M. Plasma pyridoxine deficiency is related to increased psychological distress in recently bereaved homosexual men. *Psychosom. Med.* **1998**, 60, 297-308.
10. Jung, H.Y.; Kim, D.W.; Nam, S.M.; Kim, J.W.; Chung, J.Y.; Won, M.H.; Seong, J.K.; Yoon, Y.S.; Yoo, D.Y.; Hwang, I.K. Pyridoxine improves hippocampal cognitive function via increases of serotonin turnover and tyrosine hydroxylase, and its association with CB1 cannabinoid receptor-interacting protein and the CB1 cannabinoid receptor pathway. *Biochim. Biophys. Acta Gen. Subj.* **2017**, 1861, 3142-3153.
11. McCarty, M.F. High-dose pyridoxine as an 'anti-stress' strategy. *Med. Hypotheses* **2000**, 54, 803-807.
12. Yoo, D.Y.; Kim, W.; Kim, D.W.; Yoo, K.Y.; Chung, J.Y.; Youn, H.Y.; Yoon, Y.S.; Choi, S.Y.; Won, M.H.; Hwang, I.K. Pyridoxine enhances cell proliferation and neuroblast differentiation by upregulating the GABAergic system in the mouse dentate gyrus. *Neurochem. Res.* **2011**, 36, 713-721.

13. Dakshinamurti, K.; Dakshinamurti, S.; Czubryt, M.P. (2017) Vitamin B6: Effects of deficiency, and metabolic and therapeutic functions. In: *Handbook of famine, starvation, and nutrient deprivation*, Preedy, V., Patel, V., Eds.; Springer: Cham, Switzerland, pp. 1-23.
14. Stephens, M.C.; Havlicek, V.; Dakshinamurti, K. Pyridoxine deficiency and development of the central nervous system in the rat. *J. Neurochem.* **1971**, 18, 2407-2416.
15. Stephens, M.C.; Dakshinamurti, K. Brain lipids in pyridoxine-deficient young rats. *Neurobiology* **1975**, 5, 262-269.
16. Williamson, B.; Coniglio, J.G. The effects of pyridoxine deficiency and of caloric restriction on lipids in the developing rat brain. *J. Neurochem.* **1971**, 18, 267-276.
17. Abdou, H.M.; Wahby, M.M. Neuroprotection of grape seed extract and pyridoxine against triton-induced neurotoxicity. *Oxid. Med. Cell. Longev.* **2016**, 2016, 8679506.
18. Dakshinamurti, S.; Dakshinamurti, K. Antihypertensive and neuroprotective actions of pyridoxine and its derivatives. *Can. J. Physiol. Pharmacol.* **2015**, 93, 1083-1090.
19. Hwang, I.K.; Yoo, K.Y.; Kim, D.H.; Lee, B.H.; Kwon, Y.G.; Won, M.H. Time course of changes in pyridoxal 5'-phosphate (vitamin B6 active form) and its neuroprotection in experimental ischemic damage. *Exp. Neurol.* **2007**, 206, 114-125.
20. Kuypers, N.J.; Hoane, M.R. Pyridoxine administration improves behavioral and anatomical outcome after unilateral contusion injury in the rat. *J. Neurotrauma* **2010**, 27, 1275-1282.
21. Wei, Y.; Lu, M.; Mei, M.; Wang, H.; Han, Z.; Chen, M.; Yao, H.; Song, N.; Ding, X.; Ding, J.; Xiao, M.; Hu, G. Pyridoxine induces glutathione synthesis via PKM2-mediated Nrf2 transactivation and confers neuroprotection. *Nat. Commun.* **2020**, 11, 941.
22. Jeanclos, E.; Albersen, M.; Ramos, R.J.J.; Raab, A.; Wilhelm, C.; Hommers, L.; Lesch, K.P.; Verhoeven-Duif, N.M.; Gohla, A. Improved cognition, mild anxiety-like behavior and decreased motor performance in pyridoxal phosphatase-deficient mice. *Biochim. Biophys. Acta Mol. Basis Dis.* **2019**, 1865, 193-205.
23. Bayoumi, R.A.; Smith, W.R. Some effects of dietary vitamin B6 deficiency on γ-aminobutyric acid metabolism in developing rat brain. *J. Neurochem.* **1972**, 19, 1883-1897.
24. Krishna, A.P.; Ramakrishna, T. Effect of pyridoxine deficiency on the structural and functional development of hippocampus. *Indian J. Physiol. Pharmacol.* **2004**, 48, 304-310.
25. Brown, M.J.; Beier, K. Vitamin B6 deficiency (pyridoxine). StatPearls [Internet], StatPearls Publishing, Treasure Island (FL), 2019.

26. Masisi, K.; Suidasari, S.; Zhang, P.; Okazaki, Y.; Yanaka, N.; Kato, N. Comparative study on the responses of concentrations of B_6-vitamers in several tissues of mice to the dietary level of pyridoxine. *J. Nutr. Sci. Vitaminol. (Tokyo)* **2012**, 58, 446-451.

27. Rowland, N.E.; Dunn, A.J. Effect of dexfenfluramine on metabolic and neurochemical measures in restraint-stressed ob/ob mice. *Physiol. Behav.* **1995**, 58, 749-754.

28. Franklin, K.B.J.; Paxinos, G. The mouse brain in stereotaxic coordinates. Academic Press, San Diego, 1997.

29. Yoo, D.Y.; Cho, S.B.; Jung, H.Y.; Kim, W.; Choi, G.M.; Won, M.H.; Kim, D.W.; Hwang, I.K.; Choi, S.Y.; Moon, S.M. Tat-protein disulfide-isomerase A3: a possible candidate for preventing ischemic damage in the spinal cord. *Cell Death Dis.* **2017**, 8, e3075.

30. Bowling, F.G. Pyridoxine supply in human development. *Semin. Cell Dev. Biol.* **2011**, 22, 611-618.

31. Georgieff, M.K.; Brunette, K.E.; Tran, P.V. Early life nutrition and neural plasticity. *Dev. Psychopathol.* **2015**, 27, 411-423.

32. Guilarte, T.R.; Miceli, R.C.; Moran, T.H. Developmental effects of vitamin B-6 restriction on the locomotor behavior of rats. *Brain Res. Bull.* **1991**, 26, 857-861.

33. Dakshinamurti, K.; LeBlancq, W.D.; Herchl, R.; Havlicek, V. Nonparallel changes in brain monoamines of pyridoxine-deficient growing rats. *Exp. Brain Res.* **1976**, 26, 355-366.

34. Guilarte, T.R.; Wagner, H.N. Jr.; Frost, J.J. Effects of perinatal vitamin B6 deficiency on dopaminergic neurochemistry. *J. Neurochem.* **1987**, 48, 432-439.

35. Guilarte, T.R. Effect of vitamin B-6 nutrition on the levels of dopamine, dopamine metabolites, dopa decarboxylase activity, tyrosine, and GABA in the developing rat corpus striatum. *Neurochem. Res.* **1989**, 14, 571-578.

36. Almeida, M.R.; Mabasa, L.; Crane, C.; Park, C.S.; Venâncio, V.P.; Bianchi, M.L.; Antunes, L.M. Maternal vitamin B6 deficient or supplemented diets on expression of genes related to GABAergic, serotonergic, or glutamatergic pathways in hippocampus of rat dams and their offspring. *Mol. Nutr. Food Res.* **2016**, 60, 1615-1624.

37. Glier, M.B.; Green, T.J.; Devlin, A.M. Methyl nutrients, DNA methylation, and cardiovascular disease. *Mol. Nutr. Food Res.* **2014**, 58, 172-182.

38. Gilarte, T.R. The role of vitamin B6 in central nervous system development: Neurochemistry and behavior. In: Vitamin B6 Metabolism in Pregnancy, Lactation, and Infancy. CRC Press, Boca Raton, FL, pp.77-92, 1995.

39. Qian, B.; Shen, S.; Zhang, J.; Jing, P. Effects of vitamin B6 deficiency on the composition and functional potential of T cell populations. *J. Immunol. Res.* **2017**, 2017, 2197975.

40. Cohen, S.J.; Stackman, R.W. Jr. Assessing rodent hippocampal involvement in the novel object recognition task. A review. *Behav. Brain Res.* **2015**, 285, 105-117.

41. Root, E.J.; Longenecker, J.B. Brain cell alterations suggesting premature aging induced by dietary deficiency of vitamin B6 and/or copper. *Am. J. Clin. Nutr.* **1983**, 37, 540-552.

42. Zhuo, J.M.; Praticò, D. Acceleration of brain amyloidosis in an Alzheimer's disease mouse model by a folate, vitamin B6 and B12-deficient diet. *Exp. Gerontol.* **2010**, 45, 195-201.

43. Li, P.; Zhu, M.L.; Pan, G.P.; Lu, J.X.; Zhao, F.R.; Jian, X.; Liu, L.Y.; Wan, G.R.; Chen, Y.; Ping, S.; Wang, S.X.; Hu, C.P. Vitamin B6 prevents isocarbophos-induced vascular dementia in rats through N-methyl-D-aspartate receptor signaling. *Clin. Exp. Hypertens.* **2018**, 40, 192-201.

44. Fernandez, S.P.; Muzerelle, A.; Scotto-Lomassese, S.; Barik, J.; Gruart, A.; Delgado-García, J.M.; Gaspar, P. Constitutive and acquired serotonin deficiency alters memory and hippocampal synaptic plasticity. *Neuropsychopharmacology* **2017**, 42, 512-523.

45. Teixeira, C.M.; Rosen, Z.B.; Suri, D.; Sun, Q.; Hersh, M.; Sargin, D.; Dincheva, I.; Morgan, A.A.; Spivack, S.; Krok, A.C.; Hirschfeld-Stoler, T.; Lambe, E.K.; Siegelbaum, S.A.; Ansorge, M.S. Hippocampal 5-HT input regulates memory formation and Schaffer collateral excitation. *Neuron* **2018**, 98, 992-1004.e4.

46. Kempadoo, K.A.; Mosharov, E.V.; Choi, S.J.; Sulzer, D., Kandel, E.R. Dopamine release from the locus coeruleus to the dorsal hippocampus promotes spatial learning and memory. *Proc. Natl. Acad. Sci. U.S.A.* **2016**, 113, 14835-14840.

47. Zurkovsky, L.; Bychkov, E.; Tsakem, E.L.; Siedlecki, C.; Blakely, R.D.; Gurevich, E.V. Cognitive effects of dopamine depletion in the context of diminished acetylcholine signaling capacity in mice. *Dis. Model Mech.* **2013**, 6, 171-183.

48. Sahay, A.; Scobie, K.N.; Hill, A.S.; O'Carroll, C.M.; Kheirbek, M.A.; Burghardt, N.S.; Fenton, A.A.; Dranovsky, A.; Hen, R. Increasing adult hippocampal neurogenesis is sufficient to improve pattern separation. *Nature* **2011**, 472, 466-470.

49. Kirksey, A.; Morré, D.M.; Wasynczuk, A.Z. Neuronal development in vitamin B6 deficiency. *Ann. N. Y. Acad. Sci.* **1990**, 585, 202-218.

50. Wagner, C.A.; Finberg, K.E.; Breton, S.; Marshansky, V.; Brown, D.; Geibel, J.P. Renal vacuolar H+-ATPase. *Physiol. Rev.* **2004**, 84, 1263-1314.

51. Woody, S.K.; Zhou, H.; Ibrahimi, S.; Dong, Y.; Zhao, L. Human ApoE ε2 promotes regulatory mechanisms of bioenergetic and synaptic function in female brain: A focus on V-type H+-ATPase. *J. Alzheimers Dis.* **2016**, 53, 1015-1031.

52. Zhao, W.; Gao, X.; Qiu, S.; Gao, B.; Gao, S.; Zhang, X.; Kang, D.; Han, W.; Dai, P.; Yuan, Y. A subunit of V-ATPases, ATP6V1B2, underlies the pathology of intellectual disability. *EBioMedicine* **2019**, 45, 408-421.

53. Gonda, X.; Eszlari, N.; Anderson, I.M.; Deakin, J.F.; Bagdy, G.; Juhasz, G. Association of ATP6V1B2 rs1106634 with lifetime risk of depression and hippocampal neurocognitive deficits: possible novel mechanisms in the etiopathology of depression. *Transl. Psychiatry* **2016**, 6, e945.

54. Piedrahita, D.; Castro-Alvarez, J.F.; Boudreau, R.L.; Villegas-Lanau, A.; Kosik, K.S.; Gallego-Gomez, J.C.; Cardona-Gómez, G.P. β-Secretase 1's targeting reduces hyperphosphorilated Tau, implying autophagy actors in 3xTg-AD mice. *Front. Cell. Neurosci.* **2016**, 9, 498.

55. Yang, X.; Tohda, C. Heat shock cognate 70 inhibitor, VER-155008, reduces memory deficits and axonal degeneration in a mouse model of Alzheimer's disease. *Front. Pharmacol.* **2018**, 9, 48.

56. Abisambra, J.; Jinwal, U.K.; Miyata, Y.; Rogers, J.; Blair, L.; Li, X.; Seguin, S.P.; Wang, L.; Jin, Y.; Bacon, J.; Brady, S.; Cockman, M.; Guidi, C.; Zhang, J.; Koren, J.; Young, Z.T.; Atkins, C.A.; Zhang, B.; Lawson, L.Y.; Weeber, E.J.; Brodsky, J.L.; Gestwicki, J.E.; Dickey, C.A. Allosteric heat shock protein 70 inhibitors rapidly rescue synaptic plasticity deficits by reducing aberrant tau. *Biol. Psychiatry* **2013**, 74, 367-374.

57. Fontaine, S.N.; Martin, M.D.; Akoury, E.; Assimon, V.A.; Borysov, S.; Nordhues, B.A.; Sabbagh, J.J.; Cockman, M.; Gestwicki, J.E.; Zweckstetter, M.; Dickey, C.A. The active Hsc70/tau complex can be exploited to enhance tau turnover without damaging microtubule dynamics. *Hum. Mol. Genet.* **2015**, 24, 3971-3981.

58. Jinwal, U.K.; Akoury, E.; Abisambra, J.F.; O'Leary, J.C. 3rd; Thompson, A.D.; Blair, L.J.; Jin, Y.; Bacon, J.; Nordhues, B.A.; Cockman, M.; Zhang, J.; Li, P.; Zhang, B.; Borysov, S.; Uversky, V.N.; Biernat, J.; Mandelkow, E.; Gestwicki, J.E.; Zweckstetter, M.; Dickey, C.A. Imbalance of Hsp70 family variants fosters tau accumulation. *FASEB J.* **2013**, 27, 1450-1459.
